# Visual Sequence Algorithm for Moving Object Tracking and Detection in Images

**DOI:** 10.1155/2021/3666622

**Published:** 2021-12-27

**Authors:** Renzheng Xue, Ming Liu, Xiaokun Yu

**Affiliations:** ^1^School of Computer and Control Engineering, Qiqihar University, Qiqihar, Heilongjiang 161006, China; ^2^Department of Computer Science, Heilongjiang Communications Polytechnic, Qiqihar, Heilongjiang, China

## Abstract

**Objective:**

The effects of different algorithms on detecting and tracking moving objects in images based on computer vision technology are studied, and the best algorithm scheme is confirmed.

**Methods:**

An automatic moving target detection and tracking algorithm based on the improved frame difference method and mean-shift was proposed to test whether the improved algorithm has improved the detection and tracking effect of moving targets. The algorithm improves the traditional three-frame difference method and introduces a single Gaussian background model to participate in target detection. The improved frame difference method is used to detect the target, and the position window and center of the target are determined. Combined with the mean-shift algorithm, it is determined whether the template needs to be updated according to whether it exceeds the set threshold so that the algorithm can automatically track the moving target.

**Results:**

The position and size of the search window change as the target location and size change. The Bhattacharyya similarity measure *ρ* (*y*) exceeds the threshold *r*, and the target detection algorithm is successfully restarted.

**Conclusion:**

The algorithm for automatic detection and tracking of moving objects based on the improved frame difference method and mean-shift is fast and has high accuracy.

## 1. Introduction

With the rapid development of the economy and the accelerating process of urbanization, a large number of people flock to cities, which makes the traffic safety and social security problems gradually show a complicated growth trend. Social organizations are increasingly dependent on video surveillance. However, traditional video surveillance only stores the image information collected by cameras. Normally, image information is not analyzed by traditional video surveillance, and a lot of subsequent analysis work is left to the staff. Workers working in this environment for a long time will be fatigued and inefficient. Large monitoring errors will occur, and sometimes the staff will even ignore important information, resulting in irreparable losses. In addition, video monitoring is carried out manually, which has the risk of revealing the privacy of others. When there is a safety problem, we can only watch the surveillance video, which delays the timely handling of the incident.

With the rapid development of computer hardware and software technology, the computer is running faster and faster, which makes the algorithms widely used in the fields of digital image processing, computer vision, artificial intelligence, and so on. In intelligent video surveillance, by adopting computer vision and artificial intelligence algorithms, the computer can be like a human, which can understand the content in the image and analyze and judge the content of the monitoring screen. And when an abnormal situation occurs, it can be judged in advance and reported to the staff quickly, thus helping the staff to deal with the abnormal situation in time, improving the efficiency of event handling and reducing the workload of the staff. Computer vision plays an important role in real life, and it is widely used in many fields.

Campbell proposed the optical flow method, which is characterized by an instantaneous rate when the target moves pixels in the image area. In addition, the optical flow method studies the changes of gray value in the time domain, which can be roughly divided into three categories: matching, frequency domain, and gradient. The matching-based approach is to define velocity as displacement. The method based on the frequency domain makes use of the frequency and phase information of different output filters. Although this method has high accuracy, it requires a large number of calculations [1]. In the study carried out by Defaria et al., the gradient-based method uses the gray time difference of an image sequence to calculate the speed of each pixel point. The algorithm is simple and effective, but the parameters have a great influence on the results of practical application, and there are also some problems. The advantages of the optical flow method are target detection and strong adaptability in any scene, but the disadvantages are that noise, occlusion, lighting, and other factors will affect the results, and the calculation is large, requiring good hardware support [2]. Liddo et al. proposed the mixed Gaussian model method for background modeling in the VSAM system and applied it in more complex scenes, such as shaking water waves, sudden changes in light intensity, storms, and other harsh weather conditions. As the updating speed of the dynamic background model is variable and fast, foreground objects may appear in the background; otherwise, some background objects in the background will be removed [3].

In all these application fields, the detection and tracking of moving objects is an essential link. It is an important cornerstone of subsequent target recognition and image understanding, and its results will directly affect the subsequent processing. Due to the increasing ability of computer processors to process data, many algorithms in computer vision have been realized and are widely used. Experts from all over the world have carried out a lot of research on them and put them into practical products. As an important research direction of computer vision, in the fields of video surveillance, intelligent temperature monitoring systems, and early warning systems, it has been continuously improved and applied by researchers and engineers, so it has gained more room for development. The rapidity and accuracy of moving target detection directly affect the subsequent target tracking and recognition.

Based on this, this paper puts forward the computer vision technology and different algorithms on the image moving target detection and tracking effect, which confirms the optimal algorithm scheme. Based on the improved frame difference method and mean-shift automatic detection and tracking algorithm of moving targets, check whether the improved algorithm has improved the detection and tracking effect of moving targets.

## 2. Target Tracking Algorithm

1. Tracking method based on contour: according to the energy of the contour curve, the contour curve will gradually approach the actual contour edge of the moving target under the joint action of external force and binding force. Only when the energy of the contour curve of the object edge reaches the minimum, the real contour of the moving target can be obtained. It is difficult to get the precise initialization contour of the moving object; that is, it is very difficult to initialize the boundary contour. Because it is very difficult to update the contour of the moving target in the tracking process, the effect of this method is not good when the tracking target has an occlusion problem [4].
2. Model-based tracking method: in this method, an appropriate geometric model is first established, and then model matching is carried out to realize the tracking process, and the model should be updated with timely type parameters. There are three models of moving objects: line model, 2D contour model, and 3D stereo model. The line graph model is a simple model to describe real objects; 2D projection models are very intuitive; the 3D model is relatively complex, but the model makes full use of the essential characteristics of the target object, making the tracking process more stable, especially in the complex environment. The advantage is more obvious. Compared with other tracking methods, it has higher reliability. The shortcoming lies in the large amount of calculation, which is time-consuming and difficult to meet the requirements of real-time [5].
3. Region-based tracking method: the main idea of the region tracking method is to take the common features of the connected region of the moving target as the tracking basis, such as the target texture information, color information, and so on. The selected feature information is called the reference template. The reference template determined by the first frame of the video sequence is defined as the target template, and the correlation between the target template and the candidate template in the subsequent video sequence is calculated to determine the location of the target, so as to complete the target tracking process. In practical applications, especially in complex environments, multiple features are often selected for tracking. Because the feature information based on this method is not exactly the same, the tracking accuracy and stability are better when the target object has no occlusion problem, but when the moving target has shadow and occlusion problems, the tracking processing is still difficult [6].

### 2.1. Detection and Extraction of Moving Objects by the Improved Frame Difference Method

#### 2.1.1. Single Gaussian Model to Extract Static Background

When the background environment is simple and the camera is fixed, the brightness value of the background pixels of each frame captured by the camera is near a relatively stable value, which accords with Gaussian distribution [7]. In a period of time, each pixel obeys a Gaussian distribution with mean value *μ* and standard variance *σ*, and the Gaussian distribution of each pixel is independent. Therefore, a Gaussian model can be established for each pixel as follows:

(1) $$\begin{array}{c} P\left(i_{x,y}\right)=\frac{1}{\sqrt{2\pi \delta_{x,y}^{2}}}e^{-\left({\left(i_{x,y}-\mu_{x,y}\right)}^{2}/2\delta_{x,y}^{2}\right)}, \end{array}$$

where *i*_*x*,*y*_ represents the value of a certain pixel; (*x*, *y*) indicates the coordinates of the pixel in the frame [8]; *μ*_*x*,*y*_ represents the mean value of the Gaussian model of this pixel; *δ*_*x*,*y*_ is the variance corresponding to the pixel; and *P* (*i*_*x*,*y*_) indicates the probability that the pixel value of this point is *i*_*x*,*y*_.

When no moving object enters, the value *P* (*i*_*x*,*y*_) of each pixel changes within a stable level, so it can be determined that these pixels are all background pixels [9]. When a moving target enters, the changes of pixels in the area where the target is located do not conform to Gaussian distribution, so these points can be judged as foreground pixels. The mean and standard variance of the set of pixels (*x*, *y*) in *T* frame are *μ*_*i*_ and *σ*_*i*_.

(2) $$\begin{array}{c} \mu_{i}=\frac{1}{T}\overset{T-1}{\underset{i=0}{\sum }}g_{i},\\ \sigma_{i}=\sqrt{\frac{1}{T}\sum_{i=0}^{T-1}{\left(g_{i}-\mu_{i}\right)}^{2}}, \end{array}$$

where *g*_*i*_ is the gray level of any pixel (*x*, *y*) in the *i*_th_ frame. If *G* (*x*, *y*) is the gray value of any point in the video sequence, *k* is an empirical value between 2 and 3. When |*g*_*i*+1_*x*, *y*) − *μ*_*i*_ (*x*, *y*)| ≥ *kσ*_*i*_ (*x*, *y*), it is considered as the front scenic spot [10]. When |*g*_*i*+1_*x*, *y*) − *μ*_*i*_ (*x*, *y*)| < *kσ*_*i*_ (*x*, *y*), this point is considered as the background point. The extracted background frame is marked as Dif_pM_.

#### 2.1.2. Detection of Moving Targets

In this paper, the improved five-frame difference algorithm is adopted, and two frames separated by one in five consecutive frames are used for frame difference. The obtained frame difference is “AND” with the background frame, and then the three groups of results are “OR,” so that the moving target can be detected more completely [11]. Let the five consecutive frames in the video image sequence be as follows: *p*_1_, *p*_2_, *p*_3_, *p*_4_, and *p*_5_, in which *p*_3_ is an intermediate frame. The result obtained by using two frames separated by one frame as the frame difference is as follows:

(3) $$\begin{array}{c} {\text{Dif}}_{31}=\left|p_{3}-p_{1}\right|,\\ {\text{Dif}}_{42}=\left|p_{4}-p_{2}\right|,\\ {\text{Dif}}_{53}=\left|p_{5}-p_{3}\right|. \end{array}$$

Then, the three-frame difference results dif_*p*_31__, dif_*p*_42__, and dif_*p*_53__ are, respectively, operated with the frame difference Dif_*p*_*M*__ between the intermediate frame and the background frame, which is as follows:

(4) $$\begin{array}{c} D_{1}={\text{Dif}}_{p_{31}}\cap {\text{Dif}}_{p_{M}},\\ D_{2}={\text{Dif}}_{p_{42}}\cap {\text{Dif}}_{p_{M}},\\ D_{3}={\text{Dif}}_{p_{53}}\cap {\text{Dif}}_{p_{M}}. \end{array}$$

At last, *d*_1_, *d*_2_, and *d*_3_ are OR-operated to get *d*, which is the detected moving target. There are some small holes in the moving target detected by the abovementioned improved method, which can be eliminated by the expansion and corrosion method of binary image in image morphology [12].

#### 2.1.3. Extracting Moving Objects

When a moving target is detected, the moving area of the target can be determined. In order to extract the moving target, it is necessary to remove a large number of shadows contained in the moving target area, which seriously interferes with the edge detection of the moving target [13]. Shadow detection methods are based on shadow features and geometric models. Among them, the detection method based on shadow feature is to process the image by detecting the geometric feature, color, and brightness of shadow [14]. Because the existence of shadows only affects the brightness of pixels, it does not have much influence on the color of pixels. In this paper, a shadow detection method based on HSV color space and edge detection are used to extract moving objects. First of all, the edge detection method is used to get the edge of the target moving area. Then, the shadow of the moving area of the target is detected by the HSV method, and the edge of the shadow part is obtained by edge detection. Finally, accurate target edge information can be obtained by subtracting the shadow edge from the edge of the target moving area.

There are two main approaches to visual tracking: one is the bottom-up approach, and the other is the top-down approach. This paper adopts a bottom-up approach, combined with the theory of visual computing, which can be divided into three stages: layer vision, middle layer vision, and high-level vision. Low level vision to middle level vision is an image feature description, while middle level vision to high level vision is a 2.5-dimensional description, and high level vision is a 3 dimensional description.

### 2.2. Mean-Shift Target Tracking

#### 2.2.1. Overview of Mean-Shift Algorithm

The mean-shift algorithm is a semi-automatic tracking method, which selects the moving target by manually determining the search window in the initial tracking frame [15]. The histogram distribution of the search window weighted by the kernel function is calculated, and the histogram distribution of the corresponding window of the current frame is calculated by the same method. Based on the principle of maximum similarity between the two distributions, the search window moves to the real position of the target in the direction of maximum density increase. For a finite set *a* in *n*-dimensional Euclidean space *x*, the mean-shift form at *x* ∈ *X* is as follows:

(5) $$\begin{array}{c} M_{h}\left(x\right)=\frac{\sum_{i=1}^{n}G\left(\left(x_{i}-x\right)/h\right)\omega \left(x_{i}\right)\left(x_{i}-x\right)}{\sum_{i=1}^{n}G\left(\left(x_{i}-x\right)/h\right)\omega \left(x_{i}\right)}-x. \end{array}$$

Given an initial point *x*, the kernel function *G* (*x*_*i*_), and the weight function *ωx*_*i*_, the allowable error is *ξ*. The first term on the left side of the formula is defined as *m*_*h*_ (*x*).

(6) $$\begin{array}{c} m_{h}\left(x\right)=\frac{\sum_{i=1}^{n}G\left(\left(x_{i}-x\right)/h\right)\omega \left(x_{i}\right)\left(x_{i}-x\right)}{\sum_{i=1}^{n}G\left(\left(x_{i}-x\right)/h\right)\omega \left(x_{i}\right)}. \end{array}$$

At first, calculate *m*_*h*_(*x*), and iterate the calculation result of *m*_*h*_(*x*) with *x*; if ||*m*_*h*_(*x*) − *x*|| < *ξ*, stop the iterative process.

#### 2.2.2. The Improved Frame Difference Method Is Combined with the Mean-Shift Algorithm

In order to solve the problem that mean-shift must enter the search window manually, the target edge extracted by the improved frame difference method is used to update the mean-shift search window [16]. Assuming that the bandwidth *h*, *x*_1_, *x*_2_,…, *x*_*n*_ of the initial kernel window are the sampling points included in the kernel window and *x*_0_ is the center of the target, the histogram distribution of the target is as follows:

(7) $$\begin{array}{c} {\hat{q}}_{u}=C\sum_{i=1}^{n}k\left(\left\|\frac{\left(x_{i}-x_{0}\right)}{h^{2}}\right\|\right)\delta \left[b\left(x_{i}\right)-u\right],\;u=1,2,\ldots ,m, \end{array}$$

where *k* is the kernel function; *M* is the number of eigenvalues in the feature space; *δ* is the Kronecker function; *B* (*x*_*i*_) is the characteristic value corresponding to pixel *x*_*i*_; *C* is the normalized coefficient; and *H* is the bandwidth of the kernel function. Therefore, object tracking can be simplified as finding the optimal *y*, so that *p*_*u*_ (*y*) is most similar to ${\hat{q}}_{u}$. The similarity between *p*_*u*_ (*y*) and ${\hat{q}}_{u}$ is measured by the Bhattacharyya coefficient, which is as follows:

(8) $$\begin{array}{c} \rho \left(y\right)=\sum_{u=1}^{m}\sqrt{{\hat{p}}_{u}\left(y\right){\hat{q}}_{u}}. \end{array}$$

The traditional mean-shift method does not update the template when calculating the distribution of target histogram *q*_*u*_, but always uses the template established by the initial frame, which leads to the deviation between the histogram distribution of the target template and *q*_*u*_, which easily leads to “following and losing” of the target and cannot adapt to the size change of the target. In this paper, the Bhattacharyya similarity measure coefficient *ρ* (*y*) is introduced to judge whether the template needs to be updated. When tracking the target, if the variation of *ρ* (*y*) exceeds a certain threshold value *r*, it means that the template deviation is too large. At this time, the improved frame difference method is rerun to extract the moving target, update the size and position of the tracking window, and update the histogram distribution *q*_*u*_ of the target [17].

Image segmentation is an important method in computer moving target tracking. Aiming at the vehicle tracking problem, Robert edge detection operator is used to detect the edge of the target vehicle, which greatly improves the segmentation accuracy. In addition, we propose a vehicle target segmentation strategy with the maximum interclass variance, and the experimental results show that the algorithm has a good segmentation effect.

## 3. Results

**Figure 1:**
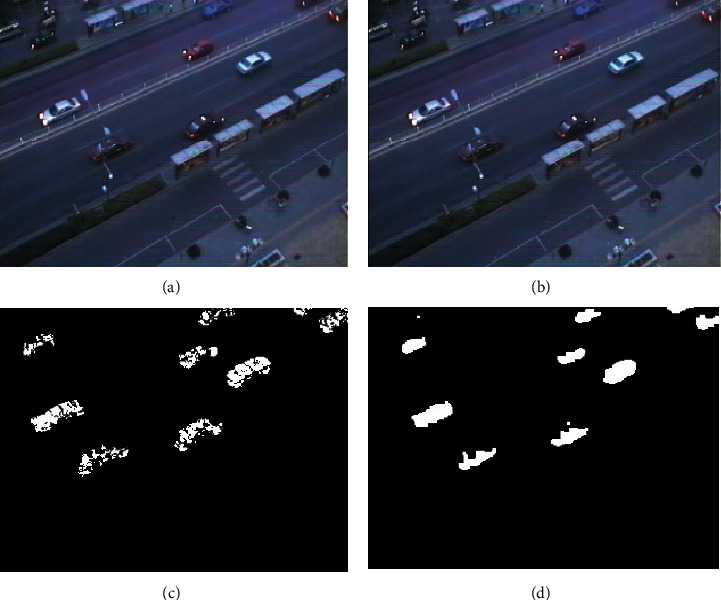
Test results of target detection. (a) 20th frame of original video, (b) 20th frame of original video, (c) traditional three-frame difference, and (d) improved results.

In this paper, a video taken indoors is used as the video source to verify the effectiveness of the algorithm. The algorithm is run on MATLAB to verify the effectiveness of the improved frame difference method by observing whether a large number of holes appear in the result of target detection. The tracking effect can be judged by observing whether the size and center position of the search box change in real time with the change of the target [18]. This is to verify that the Bhattacharyya similarity measure coefficient *ρ* (*y*) has exceeded the threshold *r* and to determine whether it has successfully restarted the target detection algorithm and updated the search window. If the target detection result is clear and no void appears, and the size and position of the tracking window of the mean-shift algorithm can be updated in real time according to the change of the target object, the validity of the algorithm can be verified. The experiment steps are as follows:

1. Read the video sequence.
2. Extract static background and five consecutive images *p*_1_, *p*_2_, *p*_3_, *p*_4_, and *p*_5_
3. Make the frame difference between the intermediate frame and the extracted static background to obtain Dif_*p*_*M*__.
4. Use the 5-frame difference algorithm mentioned above, the moving object *D* is detected.
5. The HSV transform is carried out on the detected target area, and combined with edge detection information, the shadow is eliminated, and the target frame and center point are extracted.
6. Take the target frame and center point obtained in step (5) as the template of the mean-shift algorithm.
7. Track the target frame by frame for subsequent frames, and judge whether the similarity measurement coefficient *ρ* (*y*) of Bhattacharyya exceeds the threshold. If it does not exceed the threshold, continue tracking until the end of the video frame. If it exceeds the given threshold, proceed to step (2) from the current frame, and redetect and obtain the target window. The experimental results of target detection are shown in Figures 1(a)–1(d).

**Figure 2:**
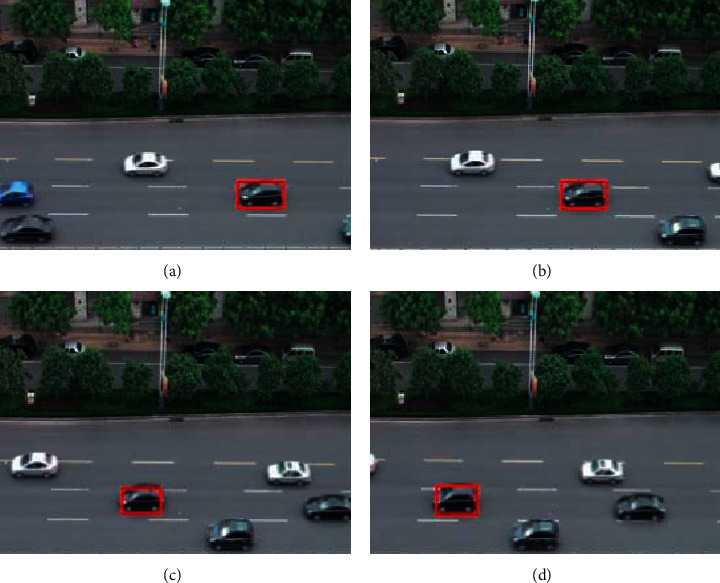
Target tracking results.

As can be seen from Figure 1, the image processed by the traditional three-frame difference method has obvious holes and a blurred target contour. The image contour obtained by the improved algorithm is clear, which shows that the algorithm has high extraction accuracy and ideal effect. The experimental results of target tracking are shown in Figures 2(a)–2(d). Figures 2(a) to Figure 2(d) are tracking effect diagrams of different frames. As shown in Figure 2, with the change of the target position and size, the position and size of the search window also change. It shows that the variation of the Bhattacharyya similarity measure coefficient *ρ* (*y*) exceeds the threshold *R*, and the target detection algorithm is successfully restarted. In the whole tracking process, there is no “follow-up” phenomenon. Besides, no one is involved in the whole process, which realizes the automatic tracking of the target.

The key of video surveillance is the detection and tracking of moving objects. Broadly speaking, this technology combines the methods of image recognition and target tracking to detect moving objects in an image sequence on the basis of analyzing static and dynamic images. In a static image, the distribution of information is constant in time and variable in space (it changes with space). And the continuous static image sequence reflects the dynamic image changes in time. Therefore, the distribution characteristics of the spatial location information density of dynamic images also change with time. Moving target detection is realized by using the time correlation between continuous frames of a static image and a dynamic image. Firstly, the moving information of the target in the dynamic image is detected, and then the following target recognition and tracking process is completed. The so-called moving target detection is to extract and separate the motion information of interest to researchers from the original video stream and separate the information features of moving objects from the background. This is regarded as the first step in the application of image analysis, intelligent monitoring, and other technologies. This paper only focuses on target tracking in visible light, and there is no indepth study on other cases. For example, targets at night will be affected by light changes, which may involve the processing of targets in infrared video images; night conditions require the system to have higher robustness, in addition to the influence of climate conditions.

## 4. Conclusion

Based on computer vision technology, in the process of detecting and tracking the moving object in the image, the improved frame difference method and mean-shift algorithm combined with the moving object detection and tracking algorithm can effectively overcome the shortcomings of the traditional frame difference method and mean-shift algorithm under the condition of static background, the position and size of the search window change as the target location and size change. The Bhattacharyya similarity measure *ρ* (*y*) exceeds the threshold *r*, and the target detection algorithm is successfully restarted. The method adopted by the program is simple, and the calculation speed is fast. It can meet the requirements of real-time detection and tracking.

## Data Availability

The data used to support the findings of this study are available from the corresponding author upon request.

## References

[1] Campbell M. D. (2019). Understanding and quantifying bias in visual fisheries surveys using advanced technology. *Fisheries*.

[2] Defaria M., Oliveiradantas D., Ruizalves M., Demedeiros B., Docarmo L. (2020). Visual acuity screening, photoscreening and dispensing of glasses with ready to clip technology. *Revista Brasileira de Oftalmologia*.

[3] Liddo A. D., Souto N. P., Plüss B. (2020). Let’s replay the political debate: hypervideo technology for visual sensemaking of televised election debates. *International Journal of Human-Computer Studies*.

[4] Karamchandani U., Erridge S., Evans-Harvey K., Darzi A., Hoare J., Sodergren M. (2020). 1190 eye-tracking technology differentiates visual gaze patterns between trainee endoscopists according to a validated objective skills assessment scale. *Gastrointestinal Endoscopy*.

[5] Blokhinov Y. B., Gorbachev V. A., Nikitin A. D., Skryabin S. V. (2019). Technology for the visual inspection of aircraft surfaces using programmable unmanned aerial vehicles. *Journal of Computer and Systems Sciences International*.

[6] Pan Y.-h. (2019). On visual knowledge. *Frontiers of Information Technology & Electronic Engineering*.

[7] Han Y., Wu A., Zhu L., Yang Y. (2021). Visual commonsense reasoning with directional visual connections. *Frontiers of Information Technology & Electronic Engineering*.

[8] Xia J.-z., Zhang Y.-h., Ye H. (2020). Supoolvisor: a visual analytics system for mining pool surveillance. *Frontiers of Information Technology & Electronic Engineering*.

[9] Lu J., Peng Y., Qi G.-J., Yu J. (2020). Guest editorial introduction to the special section on representation learning for visual content understanding. *IEEE Transactions on Circuits and Systems for Video Technology*.

[10] Gorichanaz T. (2020). Understanding and information in the work of visual artists. *Journal of the Association for Information Science and Technology*.

[11] Chegini M., Bernard J., Cui J. (2020). Interactive visual labelling versus active learning: an experimental comparison. *Frontiers of Information Technology & Electronic Engineering*.

[12] Li S.-w., Jiang Q.-b., Zhao Q.-j., Lu L., Feng Z.-l. (2020). Asymmetric discriminative correlation filters for visual tracking. *Frontiers of Information Technology & Electronic Engineering*.

[13] Lancioni G. E., O’Reilly M. F., Sigafoos J. (2019). Smartphone-based technology to support functional occupation and mobility in people with intellectual disability and visual impairment. *Advances in Neurodevelopmental Disorders*.

[14] Xie Y., Xiao J., Huang K., Thiyagalingam J., Zhao Y. (2020). Correlation filter selection for visual tracking using reinforcement learning. *IEEE Transactions on Circuits and Systems for Video Technology*.

[15] Xie Y., Xiao J., Huang K., Thiyagalingam J., Zhao Y. (2020). Correlation filter selection for visual tracking using reinforcement learning. *IEEE Transactions on Circuits and Systems for Video Technology*.

[16] Chae H.-W., Choi J.-H., Song J.-B. (2020). Robust and autonomous stereo visual-inertial navigation for non-holonomic mobile robots. *IEEE Transactions on Vehicular Technology*.

[17] Spagnolo P., Aghajan H., Bebis G. (2021). Guest editorial introduction to the special issue on large-scale visual sensor networks: architectures and applications. *IEEE Transactions on Circuits and Systems for Video Technology*.

[18] Deng L., Yang M., Liang Z., He Y., Wang C. (2020). Fusing geometrical and visual information via superpoints for the semantic segmentation of 3d road scenes. *Tsinghua Science and Technology*.

